# Replication and Injury Associated With SARS-CoV-2 in Cultured Hepatocytes

**DOI:** 10.20411/pai.v8i2.648

**Published:** 2024-02-12

**Authors:** Suman Pradhan, Susan D. Rouster, Jason T. Blackard, Gary E. Dean, Kenneth E. Sherman

**Affiliations:** 1 Department of Molecular and Cellular Biosciences, University of Cincinnati College of Medicine, Cincinnati, Ohio; 2 Division of Digestive Diseases, University of Cincinnati College of Medicine, Cincinnati, Ohio; 3 Massachusetts General Hospital-Harvard Medical School, Boston, Massachusetts

**Keywords:** COVID, SARS-CoV-2, replication, apoptosis, hepatocytes, liver, remdesivir

## Abstract

**Background:**

Liver dysfunction is one of the hallmarks of SARS-CoV-2 infection. The mechanism(s) of hepatic injury in SARS-CoV-2 infection remains controversial with some reporting viral replication and cellular injury and others suggesting lack of replication and injury due to non-cytopathogenic etiologies. To investigate this further, we evaluated SARS-CoV-2 replication in immortalized hepatic cell lines and primary hepatocytes, examined whether cell injury was associated with apoptotic pathways, and also determined the effect of the antiviral remdesivir on these processes.

**Methods:**

Immortalized hepatocyte cell lines (HepG2 and Huh7.5), as well as primary human hepatocytes, were exposed to SARS-CoV-2 at a multiplicity of infection of 0.1 PFU/mL. Viral replication was evaluated by plaque assays, immunohistochemical staining for the viral spike protein, and caspase-3 expression evaluated with and without exposure to remdesivir.

**Results:**

All hepatocyte cell lines and primary hepatocytes supported active replication of SARS-CoV-2. Significant cytopathic effect was observed by light microscopy, and caspase-3 staining supported activation of apoptotic pathways. Remdesivir abrogated infection in a dose-dependent fashion and was not independently associated with hepatocyte injury.

**Conclusion:**

Hepatocytes appear to be highly permissive of SARS-CoV-2 replication which leads to rapid cell death associated with activation of apoptotic pathways. Viral replication and hepatocytes injury are abrogated with remdesivir. We conclude that active viral replication is most likely a key contributor to liver enzyme abnormalities observed in the setting of acute SARS-CoV-2 infection.

## INTRODUCTION

Novel coronavirus SARS-CoV-2-mediated disease (COVID-19) has emerged as a major pandemic and has spread across the world with immeasurable loss of life and world economy. As of September 2023, there have been over 770 million cumulative cases of COVID-19, including nearly 7 million confirmed deaths (https://covid19.who.int/). A recent editorial documents the continued morbidity and mortality of this disease despite vaccine and antiviral tools for control [1]. Published numbers likely underestimate disease burden, as many infected patients may remain asymptomatic and can only be detected by underutilized antibody testing [2]. Severe forms of COVID-19 infection can lead to pathological inflammation, pneumonia or respiratory failure followed by multi-organ failure [3-5]. Some infected individuals are asymptomatic whilst others mount an exaggerated immune response, or ‘cytokine storm’ [6, 7] which correlates with multiple organ failure outcome [8].

Comorbidities including cardiovascular disease, diabetes, cancer, hypertension, obesity, and older age can contribute to the development of serious illness [5, 9, 10]. Individuals with chronic liver disease (CLD) often have multiple coexisting conditions which can lead to more critical illness through interactive disorders [5]. Thus, it is important to explore the relationship between underlying liver disease and SARS-CoV-2 infection to integrate appropriate therapy and improve prognosis. Liver enzyme abnormalities are a striking additional feature observed in COVID-19 patients. These abnormalities have been reported in up to 50% of patients and raise great clinical concern [11-14]. Whether or not the cytopathy of hepatocytes elicited by SARS-CoV-2 infection causes liver impairment remains unknown. Hepatocytes do express ACE2 receptors, though some have argued that a low receptor density should be associated with a lower risk of virus binding and entry. Nonetheless, as reported by Luo, et al [5] hepatoma cell lines are able to support the life cycle of SARS-CoV-2 [15-17]. Intracellular virus particles have been detected by electron microscopy within hepatocytes in liver tissue, and these findings strongly suggest SARS-CoV-2 viral replication-induced cytopathy in hepatocytes [18, 19]. Luo et al also noted that underlying liver disease can lead to increased hepatocyte ACE2 expression which may then allow access of SARS-CoV-2 into liver cells. However, some reports suggest that viral RNA was detected in only a fraction of cases and did not correlate with injury [20, 21].

To further elucidate the level and mechanism of injury seen in SARS-CoV-2 infection of hepatocytes, we evaluated replication in 3 hepatocyte-derived cell lines, including primary human hepatocytes, and described the presence of cell injury and the activation of apoptotic pathways that seem to be associated. We also demonstrated that remdesivir and other agents can reduce injury in liver cells.

## METHODS

### Cells and Propagation Media

Vero C1008 clone E6 cells (*Cercopithecus aethiops*, epithelial kidney cells) (Cat# CRL-1586), WI-38, a human diploid cell line from normal embryonic lung tissue (Cat# CCL-75), HepG2, a human liver cell line (Cat# HB-8065), and a derivative of Huh7 human liver cells (Cat# PTA-4583), were procured from ATCC and cultured in Eagle's Minimum Essential Medium (EMEM) (Cat# 30-2003) supplemented with 10% fetal bovine serum (FBS) (ATCC, Cat# 30-2002). Human plateable hepatocytes (HU 1955 (Cat# HMCPMS, Gibco, ThermoFisher Scientific USA)) were thawed and poured into a 50 mL hepatocyte thaw medium (Cat# CM7500) and centrifuged (100*g*) for 10 minutes. The supernatant was decanted, and the pellet resuspended in William's E medium containing plating supplements (Cat# CM3000). Primary hepatocytes were expanded at 37°C and 5% CO_2_ in collagen I coated transwell plates for 6 hours to overnight. After incubation, the plates were shaken to loosen debris, and the media was replaced with William's E medium containing primary hepatocyte maintenance medium. Maintenance medium was replaced every day until hepatocytes reached confluency (75%-80%) as well as during infection.

### Virus Propagation

A lyophilized ampule of human coronavirus (HCoV) strain 2019-nCoV/USA-WA1/2020 was procured from ATCC (Cat# NR-52281) and initially resuspended in EMEM supplemented with 10% FBS. Vero E6 cells were inoculated in triplicate with a dilution of 1:100 with an adsorption period of 1 hour at 37°C and 5% CO_2_, with shaking every 15 minutes. Cells were monitored for cytopathic effect (CPE) every 24 hours. Stock SARS-CoV-2 was harvested at 3 days postinfection (dpi), and supernatants were collected, clarified, aliquoted, and stored at -80°C. For replication kinetic experiments and working stock preparation, cells were seeded into 6-well plates at confluency in EMEM supplemented in 10% FBS. The next day, cells were inoculated with SARS-CoV-2 at a multiplicity of infection (MOI) of 0.1 in EMEM supplemented with 10% FBS. Supernatants were harvested at the indicated timepoints and stored at -80°C until analysis. Experiments were approved and monitored under the auspices of the University of Cincinnati Institutional Biosafety Committee. Work was performed in a BSL-3 laboratory using appropriate BSL-3 safety practices.

### Media Overlays for Plaque Assays

A 3% carboxymethyl cellulose (CMC) solution was prepared by autoclaving CMC powder (Sigma, Cat# C4888) with a magnetic stirrer in distilled H_2_O. Autoclaved powder was hydrated in 2X DMEM at 1.6% (w/v) and stirred overnight until homogenous. Double (2X) concentrated EMEM was prepared by mixing 9.48 g/L EMEM powder supplemented with 10% FBS and 2% Penicillin/Streptomycin (Penicillin 10000 Units/mL, Streptomycin 10 mg/mL). Medium was sterilized by filtration.

### SARS-CoV-2 Infection and Plaque Assay

SARS-CoV-2 plaque assay was performed as described previously [22-24]. Briefly, Vero E6 cells were seeded in 6-well plates at approximately 4 × 10^5^ cells per well and incubated until the mono-layer was 70%–80% confluent. Prior to infection, cells were washed with 1X PBS. The confluent monolayers of Vero cells were infected with 10-fold serial dilutions of SARS-CoV-2 for 1 hour at 37°C and 5% CO_2,_ with shaking at 15-minute intervals. After infection, the medium was replaced and overlaid with 3% CMC in 1:1 ratio to serum-free 2X EMEM. At 3 days postinfection, the cells were fixed with 4% formaldehyde in PBS for 2 hours at room temperature before the overlay medium was removed, and the cells were stained with 1% crystal violet solution (80% PBS and 20% methanol). Cells were then washed to reveal the plaques, and results were expressed as plaque-forming units (PFU)/mL, calculated using the formula:



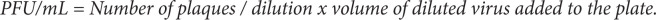


### Viral Replication Kinetics

Confluent monolayers (~70%) of Vero cells, WI-38, HepG2, Huh7.5, and HMCPMS in triplicate, were infected with SARS-CoV-2 at MOI of 0.1 and incubated for 1 hour at 37°C and 5% CO_2_ for adsorption, with shaking at 15-minute intervals. After incubation, the cells were washed twice with PBS, and fresh growth media was added. Infectious progeny virus particles released in the cell culture supernatant at various time points postinfection were quantified by plaque assay.

### Cytopathy Assay

Confluent monolayers (~70%) of Vero cells, WI-38, HepG2, Huh7.5, and HMCPMS in triplicate, were infected with SARS-CoV-2 at MOI of 0.01 for 1 hour at 37°C and 5% CO_2,_ with shaking at 15-minute intervals. After incubation, the cells were washed twice with PBS, and fresh growth media was added. Cell morphology changes by the infectious progeny virus particles were observed in an inverted microscope at various time points.

### Anti-Viral and Viral-Inhibitory Assay

Confluent monolayers (~70%) of Vero cells, WI-38, HepG2, Huh7.5, and HMCPMS were grown in 12.5 mm polyester membrane transwell plates (Fisher Scientific, Cat# 07-200-154). The apical side of the transwell plate with the cell monolayer was pre-incubated with 0.5µM and 5.0µM concentrations of remdesivir alone, virus entry receptor inhibitors/blockers (eg, 5µM quinacrine dihydrochloride [QNHC], 4µM mycophenolic acid, and 10µM Imatinib), individually or/and in combination with remdesivir for 24 hours at 37°C and 5% CO_2._ They were then infected with SARS-CoV-2 at MOI of 0.1 for 1 hour at 37°C and 5% CO_2,_ with shaking at 15-minute intervals. After the infection period, the cell monolayers were washed twice with PBS, and fresh growth media was added. The infected cells were allowed to grow for 3 days postinfection. On day 3, the cells were fixed in 4% formaldehyde solution for 8 hours. Membranes were cut out from the tran-swell and gently immersed in 30% sucrose in a 24-well tissue culture plate with the cell monolayer facing upwards overnight and until the membranes were stained for SARS-CoV-2 spike proteins and apoptosis marker caspase-3. A no-virus condition served as the negative control.

### Staining of Transwell Membrane

Transwell membranes were cut into small pieces and placed on positively charged microscope slides and blocked in blocking buffer (1% bovine serum albumin (BSA), 1% Tween20, and 22.52 mg/mL glycine) for 1 hour at room temperature. Following blocking, the membranes were incubated in SARS-CoV-2 (2019-nCoV) Spike S1 Antibody (Sino Biologicals, Cat# 40150-R007) and caspase-3 antibody (abcam, Cat# ab32351) at 1:250 dilution overnight at 4°C. The following day, glass slides were washed 3 times with sterile PBS and incubated in Alexa Fluor 488 goat anti-rabbit secondary antibody (abcam, Cat# ab150077) and Alexa Fluor 594 goat anti-rabbit secondary antibody (abcam, Cat# ab150080) at 1:500 dilution for 1 hour at room temperature in the dark. This was followed by PBS wash (X3) and staining for nuclei using 0.5 µg/mL 4’,6-diamidino-2-phenylindole (DAPI) (Invitrogen, Cat# D3571) for 5 minutes at room temperature in the dark.

### Confocal Microscopy

Stained membranes were observed under the Zeiss LSM710 LIVE Duo Confocal Microscope.

### Quantification and Statistical Analysis

All experiments were performed with 3 independent repeats. The fluorescent signal was quantified using ImageJ software. Statistical analysis was performed using GraphPad Prism version 9.

## RESULTS

### Propagation of SARS-CoV-2 *In Vitro*

The infectivity titer of the stock grown in Vero E6 cells was analyzed by plaque assay, and a virus titer of 2.1×10^6^ PFU/mL was determined.

### Comparative Kinetics of SARS-CoV-2 Replication in Different Cell Lines

To our knowledge, a detailed comparison of SARS-CoV-2 replication kinetics in distinct liver cell cultures has not been reported. Therefore, we infected 2 hepatocyte immortalized cell lines (Huh7.5 and HepG2), primary hepatocytes HMCPMS, Vero E6 cells, and a normal human embryonic lung cell line (WI-38) with the SARS-CoV-2 stock at 0.1 MOI.

**Figure 1.**
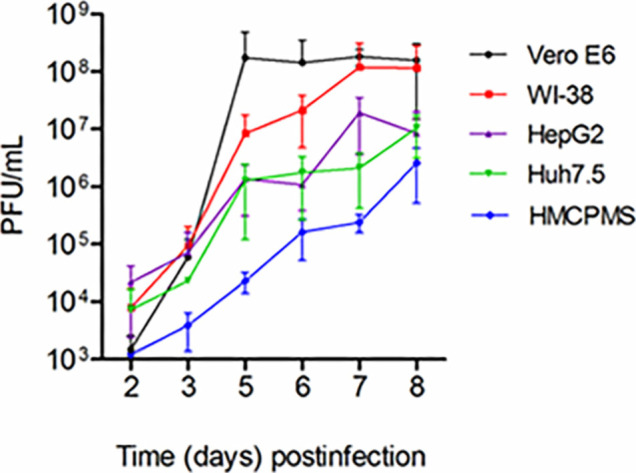
**SARS-CoV-2 virus replication**. Kidney (Vero E6), lung (WI-38), liver (HepG2, Huh7.5) and human primary hepatocytes HMCPMS were infected with SARS-CoV-2 at MOI of 0.1. About 70%-80% confluent cells were incubated with the virus for 1 hour. After incubation, wells were washed with PBS (3X) and replaced with fresh growth media. Postinfection media was collected from each well on the days indicated and analyzed by plaque assay for viral growth kinetics. (PFU/mL =plaque-forming units/mL) Error bars represent standard deviation among cell samples, n=3.

As seen in Figure 1, through 8 days postinfection, viral growth curves were similar; HepG2 and Huh7.5 were very similar to growth curves for Vero E6 and WI-38 through day 5. The lung and kidney lines showed almost 10-fold increased viral replication after day 5 postinfection before reaching a plateau at day 8. This difference was likely due to more severe cytopathic effects observed in the liver cell lines. Virus growth in primary hepatocytes (HMCPMS) was slower compared to cell lines and peaked at day 8. Plaque assays showed the presence of mainly similar plaque phenotype in all cell lines, with plaque size increasing with extension of incubation time or days postinfection (data not shown).

### Cytopathic Effects (CPE) Induced by SARS-CoV-2

**Figure 2.**
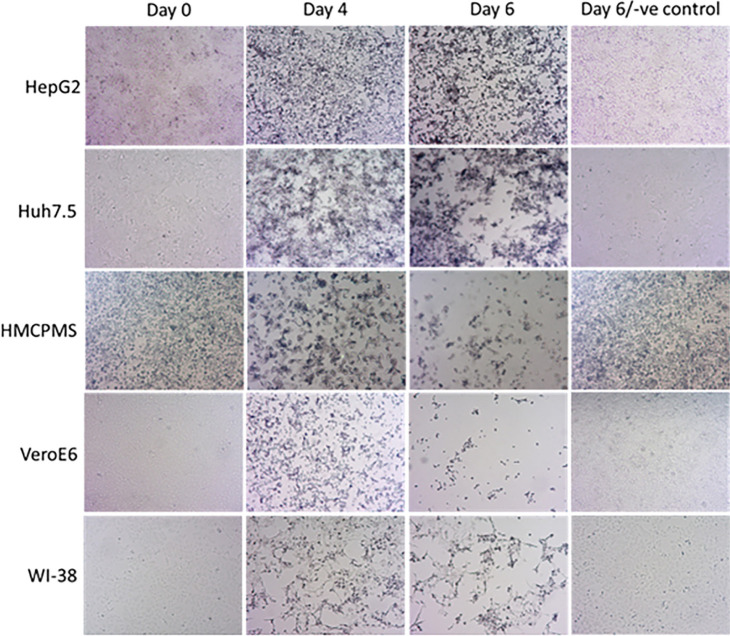
**SARS-CoV-2 causes cytopathic effect on cell monolayers**. About 70%-80% confluent cells were inoculated with SARS-CoV-2 at MOI of 0.1 plaque forming unit (PFU)/cell and incubated for 1 hour. After incubation, wells were washed with PBS (3X), replaced with fresh growth media, and monitored for cytopathic effect at the indicated timepoints. The last panel images represent negative controls at day 6. Images were collected using an inverted light microscope (10x magnification for all images).

To investigate the CPE in more detail, SARS-CoV-2-infected cell lines were analyzed by inverted light microscope. On higher magnification, plaque-like CPE was consistently observed in SARS-CoV-2-infected cells (Figure 2).

The size and number of plaques increased with the extension of the incubation time or days postinfection (data not shown). Cellular death effect observed at various days postinfection by microscopy showed an observable decrease in the number of adherent cells. Changes in cell morphology were noted to include hydropic degeneration and other cytopathic effects.

### Remdesivir Blocks SARS-CoV-2 Infection in Hepatocytes

**Figure 3.**
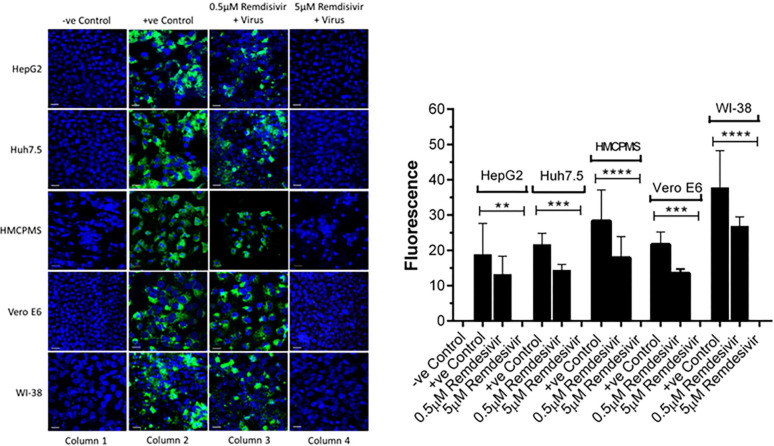
**Spike protein expression and quantification.** Confluent (70%-80%) cells were preincubated with 0.5µM and 5µM remdesivir for 24 hours in a 12.5 mm polyester membrane transwell plate. Following incubation, the cells were infected with SARS-CoV-2 at MOI of 0.1 for 1 hour, washed with PBS (3X), and replaced with complete growth media. Infected cells were further incubated for 3 days and stained for SARS-CoV-2 spike protein expression levels (A) and quantified (B). Virus only was used as positive control and no virus as negative control. (B) The fluorescent signal was quantified using ImageJ software. Statistical analysis was performed using GraphPad Prism (version 9) software, using one-way ANOVA. **P = 0.0019, ***P = 0.0003, ****P = <0.0001. Fluorescent signal was captured using Zeiss LSM710 LIVE Duo confocal microscope. Bar indicates 20µm. n=3

Cell lines were pre-incubated with 0.5µM and 5.0µM concentrations of remdesivir and infected with SARS-CoV-2 (USA-WA1/2020) at a concentration of 0.1 MOI. At 72 hours postinfection, immunostaining with antibodies against the spike protein of SARS-CoV-2 showed viral replication in all cell lines preincubated with 0.5µM remdesivir (Figure 3A, column 3). However, viral replication was completely blocked in cells preincubated with 5.0µM remdesivir (Figure 3A, column 4). The infected viral load was quantitated as an expression of spike protein expression (Figure 3B). Data suggest that SARS-CoV-2 is more infectious to primary hepatocytes compared to immortalized cell lines with higher spike protein expression observed. Statistical analysis of the quantified spike protein expression data indicated that a 0.5µM concentration of remdesivir caused reduction of the spike protein expression in all cell lines, although the reduction failed to reach statistical significance. However, a 5.0µM concentration of remdesivir resulted in total abrogation of spike protein expression.

### Cell Death and Caspase-3 Expression

**Figure 4.**
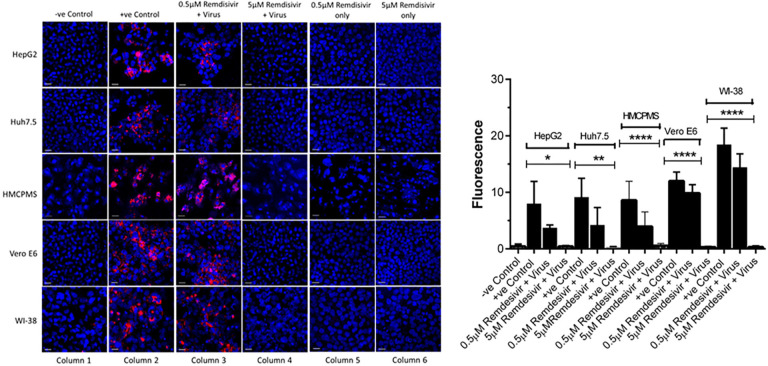
**Caspase-3 expression and quantification.** Cell lines, 70%-80% confluent, were preincubated with 0.5µM and 5µM remdesivir for 24 hours in a 12.5 mm polyester membrane transwell plate. Following incubation, the cells were infected with SARS-CoV-2 at MOI of 0.1 for 1 hour, washed with PBS (3X), and replaced with complete growth media. Infected cells were further incubated for 3 days before being stained for apoptotic marker caspase-3 (A) and quantified (B). Virus only was used as positive control and no virus as negative control. (B) The fluorescent signal was quantified using ImageJ software. Statistical analysis was done using GraphPad Prism (version 9) software, using one-way ANOVA. *P = 0.0146, **P = 0.0024, ****P = <0.0001. Fluorescent signal was captured using Zeiss LSM710 LIVE Duo confocal microscope. Bar indicates 20µm. n=3

Cell lines were pre-incubated with 0.5µM and 5.0µM concentrations of remdesivir and infected with SARS-CoV-2 (USA-WA1/2020) at a concentration of 0.1 MOI. At 72 hours postinfection, immunostaining with apoptosis cell-death marker caspase-3 antibodies indicated cell death in every cell line that was preincubated with 0.5µM remdesivir (Figure 4A, column 3). However, no cell death occurred in any cell lines preincubated with 5.0µM remdesivir indicating complete protection from SARS-CoV-2 virus infection (Figure 4A, column 4).

Quantification and statistical analysis on the expressed caspase-3 antibody indicated that cell death was reduced with the antiviral drug remdesivir at 0.5µM concentration in all cell lines. However, the reduction was not statistically significant and did not completely prevent infection, confirming the earlier result of infection with regards to spike protein expression (Figure 4B). In contrast, the 5.0µM concentration of remdesivir resulted in total and significant reduction of caspase-3 expression (Figure 4B).

### Remdesivir is Non-Toxic to Cell Lines

To further establish that apoptosis and cell death were indeed the downstream consequences of SARS-CoV-2 infection, cells treated with remdesivir alone underwent immunostaining for caspase-3 marker at 72 hours postinfection. Remdesivir at 0.5µM and 5.0µM was non-toxic to the hepatocyte cell lines studied (Figure 4A, column 5, 6) as seen by the absence of any stain for the caspase-3 apoptotic marker.

### Virus Inhibitory Drugs Block SARS-CoV-2 Infection

**Figure 5.**
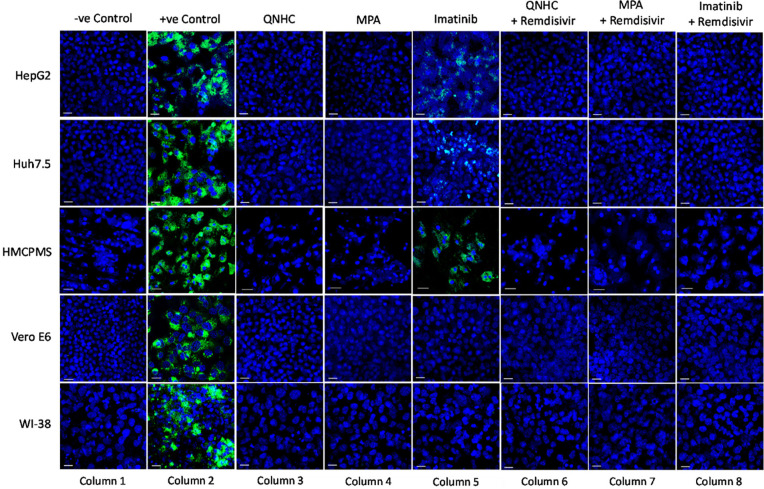
**SARS-CoV-2 infection inhibition on cell lines preincubated with antiviral and viral inhibitor commixture.** Cell lines, 70% to 80% confluent, were preincubated with 5µM quinacrine dihydrochloride (QNHC), 4µM mycophenolic acid (MPA), and 10µM imatinib and a commixture of QNHC, 5µM +5 µM remdesivir, MPA, 4µM + 5µM remdesivir, and imatinib, 10 µM + 5µM remdesivir for 24 hours in a 12.5 mm polyester membrane transwell plate. Following incubation, the wells were infected with 0.1 MOI of SARS-CoV-2 virus for 1 hour, washed with PBS (3X), and replaced with complete growth media. Infected cells were further incubated for 3 days before being stained for SARS-CoV-2 spike proteins. Virus only was used as positive control and no virus as negative control. Fluorescent signal was captured using Zeiss LSM710 LIVE Duo confocal microscope. Bar indicates 20µm. n=3

We next pre-treated the cell lines with 10μM imatinib, 4μM MPA, or 5μM QNHC for 24 hours and infected each with SARS-CoV-2 at 0.1 MOI. Drug treatment for 24 hours prior to infection resulted in significant or total reduction in viral spike protein expression. Drugs MPA and QNHC completely blocked SARS-CoV-2 infection (Figure 5, column 3, 4), whereas imatinib reduced significant levels of viral spike protein expression (Figure 5, column 5) in liver cell lines (Huh7.5, HepG2, and HMCPMS). However, imatinib totally blocked spike protein expression in kidney and lung cell lines (Figure 5, column 5).

With our previously established data of 5.0µM concentration of remdesivir, liver, kidney, and lung cell lines in combination with 10μM imatinib, 4μM MPA, or 5μM QNHC were pretreated for 24 hours prior to SARS-CoV-2 infection at 0.1 MOI. At 72 hours postinfection, immunostaining for spike protein showed no or total reduction of spike protein expression in all cell lines preincubated with QNHC plus remdesivir (Figure 5, column 6), in all cell lines preincubated with MPA plus remdesivir (Figure 5, column 7), and in all cell lines preincubated with imatinib plus remdesivir (Figure 5, column 8).

## DISCUSSION

SARS-CoV-2 continues to infect people around the world, with a global burden of more than 770 million infected people (https://covid19.who.int/). Vaccine availability, vaccine hesitancy, and the ongoing evolution and emergence of different SARS-CoV-2 variants are collectively delaying the global control of this pandemic and potential achievement of global population-level immunity [25-27]. Emerging variants of coronaviruses may continue to pose a threat to global public health.

In this study, we demonstrated that SARS-CoV-2 infects and replicates within a range of human liver cells including primary human hepatocytes. To date, there has been considerable controversy as to the nature and cause of liver enzyme abnormalities which are commonly observed in patients with COVID. Etiologic factors which have been postulated include sepsis, hypotension associated ischemia, sinusoidal thrombosis with ischemia, non-specific drug toxicity, remdesivir toxicity, and direct cytopathic effect due to viral infection. It is certainly possible that all of these mechanisms play a role in the observed outcomes. However, our data conclusively indicate that a direct viral-associated cytopathic effect modulated through caspase-mediated apoptotic pathways is likely to be the proximal cause of acute liver injury. We speculate that failure to identify infectious virus in post-mortem liver tissue reflects the transient nature of viral replication and cellular injury and does not imply lack of viral replication in the earlier stages of disease.

A morphological cytopathic death effect induced by SARS-CoV-2 was observed on day 6 post-infection by microscopy, which showed decreased adherent cells. Additional cytopathic effects including cell rounding and degeneration were observed, indicating that viral replication was causing cellular inflammation. As noted by Zupin, et al [28] the association of the SAR-CoV-2 spike protein with the plasma membrane can permit development of receptor-dependent syncytia. They also reported that cell lines expressing ACE2 binding motifs only form syncytia when co-cultured with cells that express spike protein. Buchrieser et al demonstrated that SARS-CoV-2-infected cells, expressing ACE2 or spike proteins, generated syncytia formation in Vero cells [29]. Our microscopy images revealed multiple fusion of cells after infection, but syncytia formation was not observed. However there are previous reports in other cell lines infected with SARS-CoV-2 that confirm formation of fused multinucleate cells [17].

Remdesivir (GS-5734) is an adenosine analogue and is a potent inhibitor of the RNA-dependent RNA polymerase that has been approved by the FDA for treatment of SARS-CoV-2 infection and marketed with the tradename Veklury. Product guidance indicates an increased risk of transaminase elevations, but notes “discerning the contribution of Veklury to transaminase elevations in patients with COVID-19 can be challenging.” Our data suggest that this antiviral agent does not contribute to hepatocyte injury, nor does it activate caspase-associated apoptotic pathways. This may lead clinicians to be more circumspect when treating complicated SARS-CoV-2 infected patients when liver enzyme abnormalities are present.

## CONCLUSION

Multiple hepatocyte cell lines and primary hepatocytes were highly permissive to SARS-CoV-2 replication. Injury to hepatocytes is severe and appears to be mediated via apoptotic pathways that utilize the cell death receptor family signaling network. Infected cells exhibited cell death injury as evidenced by caspase-3 staining. Replication and cellular injury are reduced by exposure to remdesivir prior to SARS-CoV-2 exposure. Our data suggest that transaminase elevations following SARS-CoV-2 infection could be due to viral replication and cellular injury and that remdesivir antiviral therapy could reduce this progression of hepatocyte damage. Remdesivir appears to inhibit replication and reduce cell injury/apoptosis in a dose-dependent manner. Hepatocyte cell lines are highly permissive for SARS-CoV-2 replication and demonstrate cytopathic injury associated with activation of apoptotic pathways. Early antiviral treatment of patients with chronic liver disease may help to limit hepatocyte injury. Remdesivir and viral cell entry inhibitors QNHC and MPA have been shown to be safe for use in humans and show efficacy in limiting the effects of SARS-CoV-2 viral replication in hepatocyte cells. The improved potency and usage of these inhibitors to reduce cellular damage due to viral replication, in light of the ongoing COVID-19 pandemic, with the increasing identification of new SARS-CoV-2 variants, remains to be determined.
